# Proton pump inhibitors may increase the risk of cisplatin-induced acute kidney injury in patients with nasopharyngeal carcinoma: a prospective cohort study

**DOI:** 10.1038/s41598-024-69821-6

**Published:** 2024-08-13

**Authors:** Haiqing Luo, Guihua Yi, Haifeng Tang, Lingli Chen, Liren Hu, Donghong Yang, Zihong Chen, Haiwen Li, Dechao Zhan, Ying Yu, Ying Zeng, Yilin Cai, Jiayuan Wu, Huafeng Liu

**Affiliations:** 1 Specialty of Head and Neck Oncology, Affiliated Hospital of Guangdong Medical University, Zhanjiang, 524002 China; 2 School of Public Health of Guangdong Medical University, Zhanjiang, 524023 China; 3 Clinical Research Service Center, Affiliated Hospital of Guangdong Medical University, Zhanjiang, 524002 China; 4 Guangdong Provincial Key Laboratory of Autophagy and Major Chronic Non-communicable Diseases, Key Laboratory of Prevention and Management of Chronic Kidney Disease of Zhanjiang City, Institute of Nephrology, Affiliated Hospital of Guangdong Medical University, Zhanjiang, 524002 China

**Keywords:** Nasopharyngeal carcinoma, Proton pump inhibitors, Chemotherapy, Acute kidney injury, Chronic kidney disease, Head and neck cancer, Oncology

## Abstract

Cisplatin is the most commonly used platinum-based treatment for nasopharyngeal carcinoma (NPC). However, its clinical application is limited owing to its nephrotoxicity and gastrointestinal reactions. Proton pump inhibitors (PPIs) have been reported to increase nephrotoxicity risk in previous studies. We aimed to evaluate whether PPIs increase cisplatin-induced nephrotoxicity in patients with NPC. In total, 295 patients were included in this prospective cohort study: 145 in the PPIs group and 150 in the non-PPIs group. All patients underwent cisplatin-based induction chemotherapy, followed by cisplatin-based concurrent chemoradiotherapy. The PPIs group received 40 mg of intravenous esomeprazole sodium for 7 days in each chemotherapy cycle. Chi-squared test and logistic regression analyses with odds ratios and 95% confidence intervals were applied to assess the association between PPIs and the risk of acute kidney injury (AKI). AKI incidence in the PPIs group was significantly higher than that in the non-PPIs group (*P* = 0.005). After adjusting for various confounders including demographic features, clinical features, and renal function indices, PPIs use was significantly associated with a higher AKI risk (odds ratio: 2.775; 95% confidence interval 1.280–6.020; *P* = 0.010). The incidences of acute and chronic kidney diseases were similar between both groups (*P* > 0.05), whereas the incidence of nausea was lower in the PPIs group than in the non-PPIs group (*P* = 0.029). This study has shown that PPIs use may increase the risk of cisplatin-induced acute nephrotoxicity in patients with NPC.

## Introduction

Head and neck cancer is the sixth most common malignant tumor worldwide, with an insidious onset. Approximately two-thirds of patients are in the middle or advanced stages when they seek medical treatment^1^. Nasopharyngeal carcinoma (NPC) is one of the most common malignant tumors of the head and neck and is prevalent in southern China and Southeast Asia^2^. Chemotherapy combined with radiotherapy is the primary treatment for NPC, especially concurrent chemoradiotherapy (CCRT), which is the first-line treatment strategy for patients with locoregionally advanced NPC^3^.

Cisplatin is a widely used anticancer drug for treating NPC. The main side effects of cisplatin include nephrotoxicity, nausea-vomiting, ototoxicity, and myelosuppression. Particularly, 20–40% of patients treated with cisplatin experience cisplatin-induced nephrotoxicity, limiting the amount of drug that can be administered^4,5^. According to the National Comprehensive Cancer Network (NCCN) guidelines on antiemesis, cisplatin is a highly emetic chemotherapy drug, and more than 90% of patients receiving cisplatin chemotherapy may develop nausea and vomiting without preventive antiemetic^6^. Chemotherapy-induced nausea and vomiting is a common clinical symptom of gastrointestinal reactions to cisplatin chemotherapy^7^. The NCCN guidelines recommend using proton pump inhibitors (PPIs) in patients receiving highly emetogenic chemotherapy. However, previous studies have demonstrated that PPIs use is associated with increased risks of acute kidney injury (AKI), chronic kidney disease (CKD), CKD progression, and end-stage renal disease (ESRD)^8–10^.

Ye et al. revealed that lansoprazole aggravated cisplatin-induced AKI in a mouse model using western blotting and real-time fluorescence quantitative polymerase chain reaction (PCR) analysis^11^. In our previous study, mice were randomly divided into different groups: control group (CON), lansoprazole 25 group (LPZ), cisplatin group (CIS), cisplatin + lansoprazole 12.5 group (CIS + LPZ12.5) and cisplatin + lansoprazole 25 group (CIS + LPZ25)^11^. Cisplatin was administered as a single dose of 18 mg/kg via intraperitoneal injection, and lansoprazole was administered at a dose of 12.5 mg/kg or 25 mg/kg 2 h before cisplatin administration. Renal tubular dilatation, tubular cell necrosis, and cast formation were observed in renal slices following cisplatin treatment. Compared with CIS, the renal injury of CIS + LPZ 12.5 and CIS + LPZ 25 increased significantly with the increase in dosage when lansoprazole was used in combination therapy. In addition, the conspicuous upregulation of serum creatinine (SCr) and blood urea nitrogen (BUN) in the CIS + LPZ 12.5 and CIS + LPZ 25 groups further confirmed this result. Based on these findings, we hypothesized that using PPIs in combination with cisplatin chemotherapy to treat NPC may further increase the incidence of AKI, thereby increasing the disease and economic burden on patients with NPC. Currently, there are few relevant studies on the effects of cisplatin-based chemotherapy combined with PPIs on renal toxicity in patients with NPC. Therefore, we conducted a prospective cohort study to evaluate whether PPIs use was associated with an increased risk of AKI in NPC patients treated with cisplatin. This study will provide a scientific basis for reasonable use of PPIs by clinicians during cisplatin chemotherapy for NPC.

## Methods

### Study design and patients

This study was conducted in accordance with the Helsinki Declaration and approved by the Institutional Review Committee of the Affiliated Hospital Guangdong Medical University (PJ2019-039). This study was prospectively registered in the Chinese Clinical Trial Registry (Date of first registration: 10/31/2020, Registration number: ChiCTR2000039569).

Written informed consent has been obtained from all participants involved in the study. Patients with biopsy-proven NPC were recruited from the Affiliated Hospital of Guangdong Medical University between November 2020 and May 2023. The inclusion criteria were: (1) age between 18 and 75 years with expected survival over 3 months; (2) diagnosis as NPC stages III–IVA based on the American Joint Committee on Cancer Guidelines 8th edition; (3) Karnofsky performance score ≥ 70; (4) receive cisplatin-based chemotherapy; and (5) renal function indicators measured before and after chemotherapy. The exclusion criteria were: (1) CKD; (2) ESRD; (3) AKI before chemotherapy; (4) use of methotrexate, mycotoxin, mitomycin-C, nitrosourea, or concurrent use of other nephrotoxic chemotherapeutic drugs; (5) previous routine use of PPIs; (6) primary malignant tumor invasion or metastasis to the kidney, ureter, bladder, or other organs; (7) allergy to the drugs used in the trial; and (8) incomplete patient information.

### Treatments

Eligible patients received three cycles of induction chemotherapy (ICT) followed by three cycles of cisplatin-based CCRT. The choice of ICT regimen was based on the clinical judgment of the attending physician. The ICT regimen included gemcitabine plus cisplatin (GP) regimen (gemcitabine 1000 mg/m^2^, cisplatin 80 mg/m^2^) and docetaxel plus cisplatin (TP) regimen (docetaxel 75 mg/m^2^, cisplatin 75 mg/m^2^) once every 3 weeks for three cycles. Radiotherapy was initiated 3 weeks after the last ICT cycle. During CCRT, cisplatin was administered concurrently with radiotherapy at 100 mg/m^2^ every 3 weeks for three cycles. According to the NCCN guidelines, PPIs should be used when receiving cisplatin-based chemotherapy in patients with pre-existing gastric diseases or those who experience a gastrointestinal reaction of more than two degrees during chemotherapy^6^. Patients were divided into PPIs and non-PPIs groups based on whether they used PPIs. The PPIs group received an intravenous drip of esomeprazole sodium (40 mg/time, once a day) starting 1 day before each cisplatin chemotherapy cycle for 7 days, for a total of six cisplatin chemotherapy cycles, including 3 ICT cycles and 3 CCRT cycles based on cisplatin. The total dose of esomeprazole sodium was 1680 mg. The non-PPIs group received chemotherapy without PPIs.

### Data collection

The following information was collected from each patient: age, sex, weight, height, body surface area (BSA), tumor stage, history of diabetes and hypertension, ICT regimen, potassium, and infusion volume. The values of SCr, eGFR, BUN, and cystatin C (CysC) before and after chemotherapy were also recorded. All patients received an antiemetic premedication with a 5-hydroxytryptamine (5-HT) 3 receptor antagonist and a neurokinin-1 receptor antagonist. Adverse effects were defined according to the Common Terminology Criteria for Adverse Events version 5.0. In order to minimize the bias caused by missing data, variables with over 20% missing data were removed from the analysis dataset, and others were duplicated using multiple imputation.

### Assessment of outcome

AKI was set as the primary outcome and was diagnosed and staged according to the kidney disease improving global outcome criteria^12^. AKI is defined as renal insufficiency caused by injury leading to changes in renal structure or function as well as meeting one of the following three conditions: (1) an increase in SCr within 48 h with the absolute value increasing by ≥ 26.5 μmol/l (0.3 mg/dl); (2) an increase in SCr by ≥ 50% (reaching 1.5 times the baseline value) within 7 days; (3) a decrease in urine volume by < 0.5 ml/ (kg ∙ h) for more than 6 h. Most patients did not have a catheter; thus, urine volume was not used to diagnose AKI. Therefore, the standard SCr was used in this study. AKI stage I was defined as an increase in SCr by ≥ 26.5 μmol/l (0.3 mg/dl) within 48 h, or SCr increase by ≥ 50% within 7 days. AKI stage II was defined as an increase in SCr level to 2.0–2.9 times the baseline value, and AKI stage III was defined as an increase in SCr levels to ≥ 3 times the baseline value, or increase of ≥ 354 μmol/l, or the initiation of renal replacement therapy. Patients who met AKI criteria for ≥ 7 days and less than 3 months after enrollment were considered to have acute kidney disease (AKD). CKD was defined as previous evidence indicating persistence of the markers of renal injury for ≥ 3 months (microalbuminuria, proteinuria > 500 mg/24 h or imaging abnormalities) or eGFR < 60 ml/min per 1.73 m^2^ for ≥ 3 months. Patients in both groups were tested for renal function indicators before and 48 h after cisplatin chemotherapy in each cycle to observe the occurrence of AKI. Patients with NPC with AKI were followed up every 3 months for 1 year to evaluate the long-term effects of PPIs on renal function in patients receiving cisplatin chemotherapy.

### Statistical analysis

The significance level (α) was defined as 0.05, and power calculations (1-β) were defined as 0.9. Based on our previous clinical results for the primary outcome, the sample size was calculated using the AKI incidence rate, and the minimum sample size was 270.

The distribution of the variables was evaluated using the Shapiro–Wilk test. Normally distributed variables were expressed as means and standard deviations and compared using the independent-samples t-test, whereas non-normally distributed variables were reported as medians with interquartile ranges and compared using the Wilcoxon rank-sum test. Categorical variables were expressed as numbers and percentages and compared using the chi-square test or Fisher’s exact test. Pre- and post-treatment comparisons in different groups were performed using the paired-samples t-test or Mann–Whitney *U* test. Logistic regression models with odds ratios (ORs) and 95% confidence intervals (CIs) were used to evaluate the effect of PPIs use on AKI risk. The crude model only included whether PPIs were used, without adjustment for confounders. In model 1, we adjusted for demographic features, including age, sex, height, weight, and BSA. In model 2, we further adjusted for clinical features, including clinical stage, ICT regimens, hypertension, diabetes, and chemotherapy hydration. In model 3, covariates were additionally adjusted for baseline renal function indices, including SCr, eGFR, BUN, and CysC. All statistical analyses were performed using R software version 4.3.1 (R Foundation for Statistical Computing, Viena, Austria). A two-sided *P* value of less than 0.05 was considered statistically significant.

## Results

### Patient characteristics

**Figure 1:**
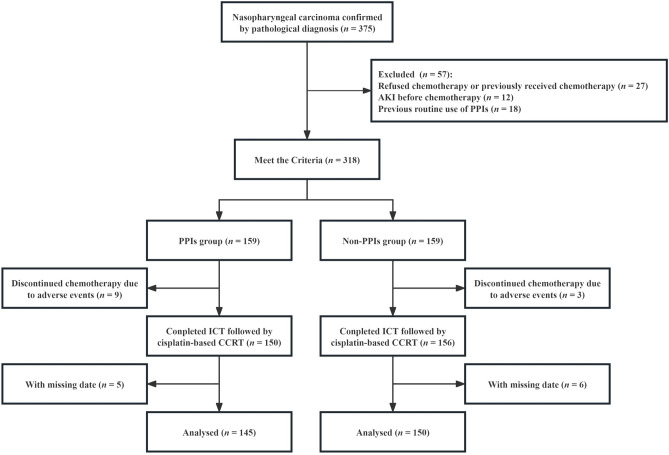
Flowchart for study patient inclusion and exclusion. *PPIs* proton pump inhibitors, *ICT* induction chemotherapy, *CCRT* concurrent chemoradiotherapy.

The study flow chart is illustrated in Fig. 1. We included 295 patients with NPC in this study: 145 patients in the PPIs group and 150 patients in the non-PPIs group. Overall, the patients had a mean age of 50.75 years, and 229 (78%) were men. One-hundred twenty (41%) patients had stage III disease, and 175 (59%) had stage IVA disease. A total of 105 (36%) patients received GP regimen for three cycles followed by CCRT, and 190 (64%) patients received TP regimen for three cycles followed by CCRT. Regarding comorbidities, a few patients in both groups were diagnosed with diabetes (9%) and hypertension (11%). In this study, patients in both groups received approximately 3,000 ml of hydration daily during chemotherapy. The baseline features of the two groups are presented in Table 1.

**Table 1:** Patient characteristics of PPIs group and Non-PPIs group.

Characteristic	PPIs group (*n* = 145)	Non-PPIs group(*n* = 150)	*P*
Gender			0.687
Male, n (%)	114 (78.6)	115 (76.7)	
Female, n (%)	31 (21.4)	35 (23.3)	
Age (years), mean (SD)	51.6 (12.1)	49.9 (11.1)	0.194
Weight (kg), mean (SD)	58.2 (10.0)	58.0 (10.0)	0.828
Height (cm), mean (SD)	164.7 (7.1)	164.2 (6.9)	0.481
BSA (m^2^), mean (SD)	1.6 (0.1)	1.6 (0.2)	0.707
Stage			0.479
III, n (%)	56 (38.6)	64 (42.7)	
IVA, n (%)	89 (61.4)	86 (57.3)	
ICT regimen			0.882
Gemcitabine plus cisplatin^a^, n (%)	51 (35.2)	54 (36.0)	
Docetaxel plus cisplatin^b^, n (%)	94 (64.8)	96 (64.0)	
Baseline renal function			
SCr (μmol/l), mean (SD)	71.6 (14.0)	71.1 (14.3)	0.755
eGFR (ml/min per 1.73 m^2^), mean (SD)	99.6 (14.0)	101.0 (14.2)	0.400
BUN (mg/dl), mean (SD)	11.6 (3.0)	12.1 (3.5)	0.147
CysC (mg/l), mean (SD)	0.8 (0.2)	0.8 (0.2)	0.945
Potassium (mmol/l), mean (SD)	4.1 (0.3)	4.2 (0.3)	0.146
Chemotherapy hydration (ml), mean (SD)	3071.7 (196.7)	3037.7 (215.1)	0.157
Hypertension, n (%)	18 (12.4)	14 (9.3)	0.395
Diabetes, n (%)	15 (10.3)	12 (8.0)	0.485

*PPIs* proton pump inhibitors, *BSA* body surface area, *ICT* induction chemotherapy, *CCRT* concurrent chemoradiotherapy, *SCr* serum creatinine, *eGFR* estimated glomerular filtration rate, *BUN* blood urea nitrogen, *CysC* cystatin C.

^a^Cisplatin dose of 80 mg/m^2^, ^b^Cisplatin dose of 75 mg/m^2^.

### Primary outcome

**Figure 2:**
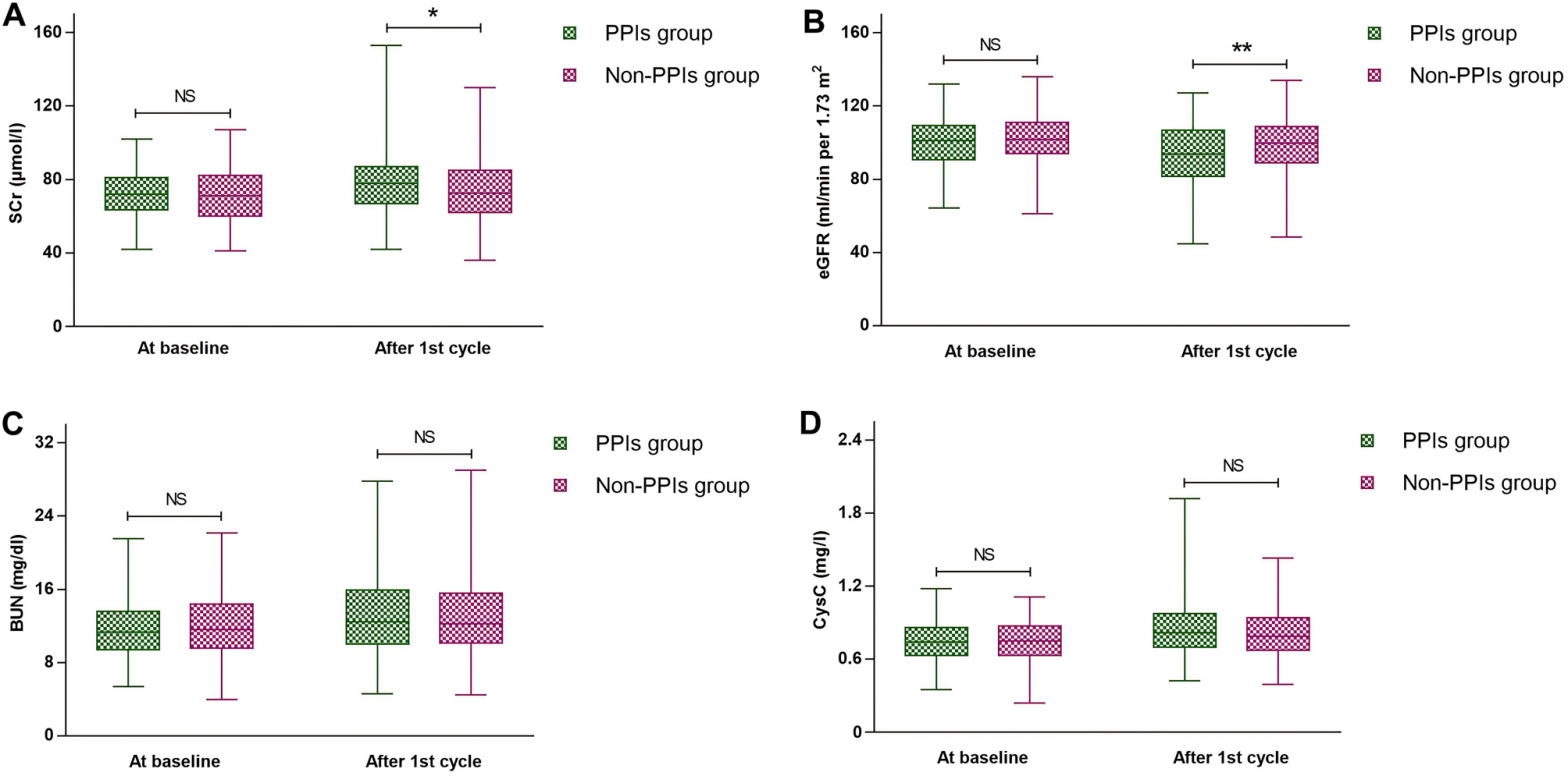
Variation in different renal function indicators before and after the first cycle of chemotherapy in two groups. (**A**) serum creatinine (SCr). (**B**) estimated glomerular filtration rate (eGFR). (**C**) Blood urea nitrogen (BUN). (**D**) cystatin C (CysC). *P < 0.05; and **P < 0.01. *PPIs* proton pump inhibitors, *NS* not significant.

The incidence of AKI was 19.3% (28/145) in the PPIs group and 8.0% (12/150) in the non-PPIs group, with a significant difference (*P* = 0.005; Table 2). In addition, most patients with AKI in both groups were at stage 1, with 27 patients in the PPIs group and 11 patients in the non-PPIs group, respectively. Only one patient in each groups had stage II AKI, and no stage III AKI was present in either group. A significant difference was observed in the severity of AKI between the two groups (*P* = 0.005; Table 2). Most patients had increased values of SCr, BUN, and CysC and decreased values of eGFR after cycle 1 of chemotherapy compared with those before chemotherapy. A comparison of renal function before and after chemotherapy is illustrated in Fig. 2. After chemotherapy, the average SCr level in the PPIs group was significantly higher than that in the non-PPIs group (*P* = 0.016; Fig. 2A), and the average eGFR in the PPIs group was significantly lower than that in the non-PPIs group after chemotherapy (*P* = 0.004; Fig. 2B). While no significant differences were observed in the average values of BUN and CysC between the two groups after chemotherapy (*P* > 0.05), the values in the PPIs group tended to be lower than those in the non-PPIs group (Fig. 2C,D).

**Table 2:** Primary and secondary outcomes of the study: incidence and severity of AKI, incidence of AKD and CKD, and incidence of nausea and vomiting.

	Total (*n* = 295)	PPIs group (*n* = 145)	Non-PPIs group (*n* = 150)	*P*
Primary outcome				
Incidence of AKI, n (%)	40 (13.6)	28 (19.3)	12 (8.0)	0.005
Severity of AKI, n (%)				0.005
1	38 (12.9)	27 (18.6)	11 (7.3)	
2	2 (0.7)	1 (0.7)	1 (0.7)	
3	0	0	0	
Secondary outcome				
Incidence of AKD, n (%)	12 (4.1)	8 (5.5)	4 (2.7)	0.215
Incidence of CKD, n (%)	7 (2.4)	4 (2.8)	3 (2.0)	0.964
Gastrointestinal toxicity				
Nausea				0.029
Grade 1–2, n (%)	151 (51.2)	66 (45.5)	85 (56.7)	
Grade 3–4, n (%)	6 (2.0)	2(1.4)	4 (2.7)	
Vomiting				0.140
Grade 1–2, n (%)	112 (40.0)	50 (34.5)	62 (41.3)	
Grade 3–4, n (%)	6 (2.0)	2 (1.4)	4 (2.7)	

*AKI* acute kidney injury, *AKD* acute kidney disease, *CKD* chronic kidney disease, *PPIs* proton pump inhibitors.

### Secondary outcomes

The difference in the incidences of AKD and CKD between the two groups was not significant (*P* > 0.05; Table 2). Additionally, the incidence of nausea was significantly lower in the PPIs group than in the non-PPIs group (*P* = 0.029). However, no significant difference was observed in the incidence of vomiting between the two groups (Table 2).

### Logistic regression analysis

Factors associated with AKI occurrence are presented in Table 3. In univariate analysis, factors significantly associated with the occurrence of AKI were the use of PPIs (*P* = 0.006), age 45 years (*P* = 0.013), comorbid diabetes (*P* = 0.003), baseline SCr value (*P* = 0.004), baseline eGFR value (*P* = 0.013), and baseline CysC value (*P* = 0.021). In the multivariate analysis, factors significantly associated with the occurrence of AKI were the use of PPIs (*P* = 0.013), age ≥ 45 years (*P* = 0.023), comorbid diabetes (*P* = 0.009), and baseline SCr value (*P* = 0.011).

**Table 3:** Univariate and multivariate logistic regression for AKI.

Variables	Univariate		Multivariate	
	Odds ratio (95% CI)	*P*	Odds ratio (95% CI)	*P*
PPIs				
No	Reference			
Yes	2.752 (1.340–5.652)	0.006	2.584 (1.219–5.481)	0.013
Age (years)				
< 45	Reference			
≥ 45	3.439 (1.300–9.096)	0.013	4.258 (1.218–14.888)	0.023
Sex				
Male	Reference			
Female	0.346 (0.118–1.010)	0.052		
Stage				
III	Reference			
IVA	0.815 (0.416–1.595)	0.550		
ICT regimen				
Gemcitabine plus cisplatin	Reference			
Docetaxel plus cisplatin	1.030 (0.513–2.072)	0.933		
Diabetes				
No	Reference			
Yes	3.823 (1.580–9.247)	0.003	3.724 (1.386–10.006)	0.009
Hypertension				
No	Reference			
Yes	1.554 (0.596–4.051)	0.367		
Baseline renal function				
SCr (μmol/l)	1.037 (1.012–1.063)	0.004	1.067 (1.015–1.121)	0.011
eGFR (ml/min per 1.73 m^2^)	0.971 (0.948–0.994)	0.013	1.049 (0.995–1.107)	0.075
BUN (mg/dl)	1.092 (0.989–1.205)	0.083		
CysC (mg/l)	11.408 (1.435–90.698)	0.021	4.011 (0.304–52.895)	0.291
Chemotherapy hydration (ml)	1.000 (0.999–1.002)	0.579		

*AK*I acute kidney injury, *CI* confidence interval, *PPIs* proton pump inhibitors, *ICT* induction chemotherapy, *CCRT* concurrent chemoradiotherapy, *SCr* serum creatinine, *eGFR* estimated glomerular filtration rate, *BUN* blood urea nitrogen, *CysC* cystatin C.

We analyzed the relationship between the use of PPIs and the occurrence of AKI using a logistic regression model after adjusting for various confounding factors (Table 4). A crude univariate logistic regression analysis model revealed that the combined use of PPIs during cisplatin chemotherapy was significantly associated with the incidence of AKI, with an OR of 2.752 (95% CI 1.340–5.652, *P* = 0.006). After adjusting for a series of confounders, multivariate analyses indicated a significant effect of PPIs administration on AKI incidence (OR 2.775, 95% CI 1.280–6.020, *P* = 0.010) in the fully adjusted model.

**Table 4:** Associations between PPIs uses and the risk of AKI by Logistic regression models.

Model	Odds ratio (95% CI)	*P*
Crude model	2.752 (1.340–5.652)	0.006
Model 1^a^	2.492 (1.192–5.210)	0.015
Model 2^b^	2.490 (1.177–5.269)	0.017
Model 3^c^	2.775 (1.280–6.020)	0.010

^a^Model 1 was adjusted for demographic features, including age, sex, height, weight, and BSA.

^b^Model 2 was additionally adjusted for clinical features, including clinical stage, induction chemotherapy regimens, hypertension, diabetes, and chemotherapy hydration.

^c^Model 3 was additionally adjusted for renal function indices, including SCr, eGFR, BUN, and CysC.

## Discussion

Chemotherapy is important in treating NPC, and platinum-based chemotherapy is recognized as the first-line treatment. Cisplatin, one of the most common chemotherapeutic drugs, is widely used to treat NPC^13,14^. While cisplatin provides a substantial survival benefit for patients with NPC, patient adherence to treatment is reduced because of its adverse side effects, including nephrotoxicity, gastrointestinal toxicity, and bone marrow toxicity^15^. The use of cisplatin is often limited owing to the development of nephrotoxicity, with studies indicating an incidence between 20 and 30%^16^. Due to its nephrotoxic side effects, hydration and diuretics are often required during and before cisplatin administration in chemotherapy to prevent renal dysfunction^17^. However, AKI still occurs in some patients receiving hydration therapy, which affects the efficacy and prognosis of chemotherapy. The pathophysiology of cisplatin-induced AKI is highly complex and is associated with cellular uptake and efflux, oxidative stress, endoplasmic reticulum stress, inflammatory responses, apoptosis, necrosis, and autophagy^5^. AKI is a well-defined risk factor for CKD, and patients who develop AKI have a several-fold increased risk of developing CKD^18^. Renal tubulointerstitial fibrosis can occur during repeated episodes of AKI induced by cisplatin^19^, and cisplatin-induced AKI can lead to CKD.

In addition, gastrointestinal toxicity is a common complication of cisplatin chemotherapy. Chemotherapy drugs can stimulate the gastrointestinal mucosa and cause the significant release of 5-HT from intestinal enterochromaffin cells of the gastrointestinal mucosa to act on 5-HT3 receptors at the end of the gastrointestinal vagus fibers, resulting in an increased afferent impulse of the vagus nerve fibers and abnormal excitation of the vomiting center, leading to gastrointestinal reactions. Additionally, chemotherapeutic drugs and their metabolites can act directly on chemoreceptors, which send excitatory nerve impulses to the vomiting centers. Various neurotransmitters and receptors are involved in the vomiting reactions, such as 5-HT 3, dopamine receptors, acetylcholine, and NK-1 receptors^20^. Therefore, 5-HT 3 receptor antagonists, NK-1 receptor antagonists, glucocorticoids, general antiemetic drugs, PPIs, and H_2_ receptor antagonists are conventionally used to prevent nausea and vomiting. PPIs are commonly used to inhibit gastric acid secretion and protect the gastric mucosa and are commonly used clinically to reduce gastrointestinal reactions in patients during chemotherapy. Uwagawa et al. discovered that patients receiving chemotherapy had a high incidence of gastroesophageal reflux disease and that rabeprazole significantly improved the symptoms of gastroesophageal reflux disease caused by chemotherapy^21^. Wang et al. revealed that intermittent high-dose PPIs enhanced the antitumor effect of chemotherapy in patients with metastatic breast cancer without additional evidence of toxicity by reducing tumor acidity and overcoming acid-related chemoresistance^22^. In addition, some studies have also revealed that PPIs have antitumor effects on other tumors^23,24^.

PPIs are widely prescribed to reduce gastric acidity; however, over 50% of PPIs prescriptions are inappropriate or unnecessary. Additionally, several studies have demonstrated that PPIs use increases the risk of AKI, CKD, and ESRD^25–27^. Hart et al. studied the relationship between the use of PPIs and the risk of developing AKI and CKD^25^. In the AKI cohort, the incidence of AKI was significantly higher than that in non-exposed individuals. In the CKD cohort, the incidence of CKD was significantly higher than in patients who did not use PPIs. Xie et al. established a cohort study, in which enrolled patients were treated with acid inhibitors, and revealed that PPIs use was associated with an increased risk of chronic renal outcomes^26^. Klatte et al. found that people using PPIs had an increased risk of doubling creatinine levels and a 30% or more decrease in eGFR, which were associated with AKI and ESRD^27^. PPIs-acute interstitial nephritis (AIN) is the pathological manifestation of AKI caused by PPIs. Several studies have demonstrated that PPIs can cause AIN; however, the pathogenic mechanism of PPIs-AIN remains unclear. Most researchers believe that this is a specific immune-mediated response^28,29^. PPIs and/or their metabolites may be deposited in the renal tubular interstitium and bind to the normal components of the renal tubular basement membrane, acting as haptens or directly stimulating T cells to mediate AIN. In addition, PPIs can induce antibody production and deposition in the renal interstitium in the form of circulating immune complexes. Acute inflammation and injury of the renal tubulointerstitial tissue caused by PPIs-AIN can lead to chronic tubulointerstitial fibrosis, scar formation, and tubular atrophy, ultimately leading to CKD if PPIs are not stopped and treated promptly^30^.

There are currently few studies on the effects of cisplatin-based chemotherapy combined with PPIs on renal toxicity in patients with NPC. Further, the effects of AKI on long-term renal function in patients with NPC are poorly understood. We explored the association between PPIs use and AKI occurrence during cisplatin-based chemotherapy in patients with NPC. The incidence of AKI was significantly higher in the PPIs group than in the non-PPIs group (19.3% vs. 8.0%, *P* = 0.005), suggesting that PPIs may increase the incidence of AKI in patients with NPC. PPIs have been confirmed to synergistically aggravate cisplatin-induced AKI in vitro*.* Ye et al. discovered that lansoprazole up-regulated necroptotic biomarkers, including receptor-interacting protein kinase (RIPK) 1, phosphorylated receptor-interacting protein kinase (p-RIPK) 3, and phosphorylated mixed lineage kinase domain-like (p-MLKL) to induce tubular cell death via the tumor necrosis factor (TNF)-α-mediated RIPK1/RIPK3/MLKL pathway, indicating that necroptosis and inflammation aggravate cisplatin-induced AKI^11^. Some studies have shown that necroptosis promotes inflammation, the inflammatory cytokine TNF similarly induces necroptosis, indicating a potential positive feedback loop between inflammation and necroptosis^31,32^. These negative effects can lead to irreversible kidney damage. Similarly, an in vitro study demonstrated that omeprazole increased the expression of the tubular cell injury marker neutrophil gelatinase associated lipocalin (NGAL) and the oxidative stress marker Heme-Oxygenase-1 to promote cell death, supporting its nephrotoxic potential^33^.

Cisplatin-related gastric damage is usually limited to superficial erosions, mostly in the antral region^34^. Cisplatin blocks acetylcholine release in smooth muscle cells and induces pyloric spasms and accumulation of hydrochloric acid and enzymes in the stomach, causing mucosal injury^35^. PPIs are the most effective agents for treating gastric acid-related disorders and preventing mucosal damage. Sartori et al. conducted two sequential randomized trials to assess the use of PPIs as prophylactic agents in chemotherapy^36,37^. The first trial demonstrated that only 19% of patients receiving omeprazole had erosion or gastritis, compared with 47% of patients in the placebo group^36^. The second trial reported that omeprazole was significantly superior to placebo regarding endoscopic results and symptom control^37^. The results of the two trials were similar, demonstrating the overall benefits of omeprazole for mucosal protection and symptom relief. However, PPIs have no significant pharmacological gastroprotective effect in patients with cancer treated with cisplatin^38,39^, and further research is needed. Our results revealed that the incidence of nausea in the PPIs group was significantly lower than that in the non-PPIs group (*P* = 0.029), suggesting that PPIs may reduce gastrointestinal reactions in patients receiving cisplatin chemotherapy.

In our study, patients aged ≥ 45 years (OR 4.258, 95% CI 1.218–14.888), high baseline SCr level (OR 1.067, 95% CI 1.015–1.121), and comorbid diabetes (OR 3.724, 95% CI 1.386–10.006) were significantly more susceptible to cisplatin-induced AKI. Patients with AKI were older (87.5% > 45 years) than those without AKI (67.5% > 45 years). Additionally, in older patients, the renal interstice is more vulnerable to damage owing to compromised peritubular blood flow, which permits a longer exposure time between the medication and the interstice^40^. This finding is consistent with the results of previous trials and clinical studies^41,42^. The baseline SCr level, the main diagnostic test for AKI, was strongly related to the development of cisplatin-induced nephrotoxicity. Liu et al. constructed a predictive model that included baseline SCr risk factors to identify patients susceptible to cisplatin-induced nephrotoxicity^43^. Moreover, comorbid diabetes was an independent risk factor for cisplatin-induced AKI in our study. Mathe et al. reported that diabetes mellitus could result in severe microangiopathy and interstitial inflammation, increasing tissue susceptibility and further enhancing the risk of cisplatin-induced AKI in patients with lung cancer^44^. However, the underlying pathophysiological mechanism by which comorbid diabetes aggravates cisplatin-induced AKI in patients with NPC requires further exploration.

Our results suggest that combining cisplatin chemotherapy and PPIs in patients with NPC may further increase the incidence of AKI. In this study, after adjusting for confounding factors, there was a significant correlation between the combined use of PPIs during cisplatin chemotherapy and the occurrence of AKI. This aligns with previous in vivo experimental mice study, which revealed that lansoprazole increasing tubular necroptosis via TNF-α-mediated RIPK1/RIPK3/MLKL pathway further aggravates cisplatin-induced AKI^11^. Lansoprazole aggravated the renal tubular injury in mice caused by cisplatin, resulting in increased SCr and BUN. Compared with previous mouse models, the degree of renal injury in our clinical trial was lower because only SCr was high and BUN was not, which may also be related to the high doses of cisplatin and PPIs used in the animal experiments.

However, our results are different from those reported by others earlier^45–47^. The study by Ikemura et al., investigated co-administration of clinical doses of PPIs protected kidney function in patients receiving cisplatin and fluorouraci^45,46^. This discrepancy may be explained by the retrospective nature and the unbalanced sample size of the above study, which cannot adjust for all confounding variables and inevitably leads to bias. In addition, due to the different renal function evaluation time points compared to our study, the incidence of acute kidney injury and chronic kidney disease in the above study had not been reported. Ghonaim et al. reported that Pantoprazole, as promising cisplatin-nephrotoxic protective agents for treating patients with head and neck cancer, effectively ameliorated nephrotoxicity^47^. However, a clinical intervention trial reported that pantoprazole did not protect children with osteosarcoma who had received cisplatin, methotrexate, or doxorubicin against cisplatin nephrotoxicity^48^. The study did not meet the planned endpoints and revealed that PPIs combined with cisplatin did not ameliorate nephrotoxicity, which supports our findings. The differences in these results may be due to regional and ethnic differences, differences in the age of the participants, a lack of a separate non-PPIs group, different doses and administration patterns of PPIs, and different types of cancer and chemotherapy regimens used.

Despite the significant contributions of our study, several limitations warrant consideration. First, all participants in this study were Asian, which could introduce bias due to the homogeneity of the population sample. Second, the sample size is small and patients might be highly selected. Third, this study had a relatively short follow-up period of 12 months. Thus, we could not observe the long-term effect of PPIs on renal function among the NPC patients. Finally, these results stem from a single-center prospective cohort study, and since observational studies were unable to establish causal relationships, their generalizability requires further evaluation. Acknowledging these limitations, a multi-center, large-scale randomized controlled trial is needed to further validate our findings.

## Conclusions

Our study revealed that combining cisplatin chemotherapy and PPIs in patients with NPC may further increase the incidence of AKI. Additionally, PPIs can be appropriately used in cisplatin chemotherapy to prevent and treat gastrointestinal reactions caused by chemotherapeutic drugs. Our study provides strong evidence supporting the regulation of the rational use of PPIs and scientific chemotherapy drug dispensing strategies for NPC specialists.

## Data Availability

The data presented in this study are available on reasonable request from the corresponding author.
